# Dr. A. W. Calhoun

**Published:** 1872-03

**Authors:** 


DR. A. W. CALHOUN.—In this issue of the JOURNAL, we add the name of Dr. A. W. Calhoun, of Newnan, Georgia—now in Berlin, Prussia—to our list of regular contributors, having had assurances from him that the article published in this number is the first of a series which may be expected from him. While not neglecting the general interests of his profession, he is devoting himself largely, during his European residence, to surgery, and especially to the diseases of the eye and ear; and with fine intelligence, both general and professional, and with great industry, we have no doubt of a successful professional career upon his return to the United States.

